# Syntheses and Characterization of the First Cycloheptatrienyl Transition-Metal Complexes with a M-CF_3_ Bond

**DOI:** 10.3390/molecules26226838

**Published:** 2021-11-12

**Authors:** Christos Lampropoulos, Gabriel Rashad, Chris Douvris

**Affiliations:** 1 DrL Consultations, Jacksonville, FL 32257, USA; DrL.Consultations@gmail.com; 2 Florida State College at Jacksonville, Jacksonville, FL 32202, USA; 3 James Weldon Johnson MS, Duval County Public Schools, Jacksonville, FL 32207, USA; 4 Theobald Science Center, Department of Biological and Chemical Sciences, New York Institute of Technology, Old Westbury, NY 11568, USA; grashad@nyit.edu

**Keywords:** trifluoromethyl group, molybdenum complexes, cycloheptatrienyl ligand

## Abstract

The organometallic chemistry of metal complexes with organocyclic ligands of higher than five hapticity is much more lacking than the chemistry of metal complexes with η^5^-cyclopentadienyl ligands, which has been explored in considerable depth, resulting in novel advances. The main reason for this is stability. In particular, reports indicate that (η^7^-C_7_H_7_)ML_n_ complexes are considerably less stable than analogous (η^5^-C_5_H_5_)ML_n_. In perfluoroalkyl metal chemistry, there is currently no reported (η^7^-C_7_H_7_)ML_n_ derivative, whereas a number of alkylated ones are known and important conclusions have been drawn about their stability. Responding to this void, and using Morrison’s trifluoromethylating reagent, the present study reports the synthesis and characterization of the first cycloheptatrienyl molybdenum complexes bearing the trifluoromethyl moiety; (η^7^-C_7_H_7_)Mo(CO)_2_CF_3_ (**I**), and (η^7^-C_7_H_7_)Mo(CO)(PMe_3_)CF_3_ (**II**) and discusses their low thermal instability.

## 1. Introduction

The interest in the chemistry of the trifluoromethyl group is both theoretical and industrial as the demand for compounds containing CF_3_ moieties is high due to their applications in advanced materials, pharmaceuticals, agrochemicals, fluorous chemistry, and medicine [1,2]. In organometallic chemistry, despite the interest, the number of transition metal complexes bearing perfluoroalkyl ligands is small. This is especially evident if their number is compared with the that of the alkyl counterparts [3]. The plethora of known alkylated transition metal complexes and the efficiency of their preparation methods has made possible several extremely important advances in both theoretical and practical application chemistry of the C-H bond, with most notable being the study of C-H activation using transition metal catalysts [4,5]. Parallel to this, similar advances in the perfluoroalkyl chemistry could be envisioned with establishing efficient routes for transition metal alkylated complexes [6,7].

With a focus on the trifluoromethyl group, the most important of fluorocarbons, we set out to explore the synthesis and characterization of trifluoromethylated metal complexes with the metal having hapticity greater than five. It is well known that systems of (η^5^-C_5_H_5_)ML_n_ (M = transition metal, L_n_ = n-ligands) are ubiquitous in organometallic chemistry and as reports suggest of much higher stability than analogous systems with carbocyclic ligands of with higher hapticity, such as (η^7^-C_7_H_7_)ML_n_ [8]. An illustrative example is the (η^7^-C_7_H_7_)Mo(CO)_2_CH_3_, which chromatography is at −78 °C for its isolation, and it is reported to rapidly decompose even under inert atmosphere at ambient temperature [9]. In contrast to this, the complex (η^7^-C_5_H_5_)Mo(CO)_3_CH_3_ is indefinitely air stable and melts at 145 °C without decomposition [10,11].

Trifluoromethyl metal complexes are predicted to be more stable than their methylated counterparts. as the back donation from the π* orbital increases the strength to the metal to carbon bond [3]. Considering this fact, as well as the intriguing instability of the reported methylated η^7^-cycloheptatrienyl complexes, we set out to explore the synthesis and stability of analogous trifluoromethylated species. As a result, we report herein the synthesis and characterization of the first η^7^-cycloheptatrienlyl trifluoromethyl molybdenum complexes, (η^7^-C_7_H_7_)Mo(CO)_2_CF_3_ (**I**) and (η^7^--C_7_H_7_)Mo(CO)(PMe_3_)CF_3_ (**II**). As we will describe, the two new complexes are of extreme thermal instability, a fact that necessitates the use of low temperature chromatography for their purification.

## 2. Results

**Scheme 1:**
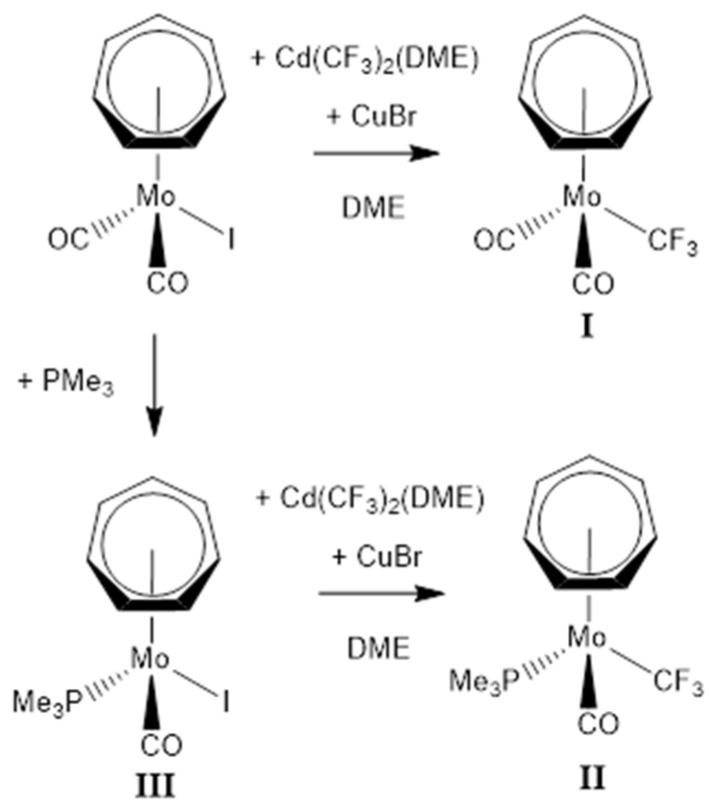
Synthesis of complexes **I** and **II**.

Upon choosing (η^7^-C_7_H_7_)Mo(CO)_2_I as the precursor for the trifluoromethylation reaction, this was initially reacted with a number of nucleophilic trifluoromethylating reagents, including CF_3_I/Cu in DMF, which is known to generate CuCF_3_ in situ [12], and (CH_3_)_3_SiCF_3_/AgF, which is known to generate AgCF_3_ in situ [13], at ambient and elevated temperatures; however, no formation of a Mo-CF_3_ bond was observed, as monitored by ^19^F NMR spectroscopy. In a reaction maintained at −20 °C between (η^7^-C_7_H_7_)Mo(CO)_2_I, and Morrison’ reagent, Cd(CF_3_)_2_(DME), (DME = dimethoxyethane), a resonance at δ −0.2 ppm, indicative of a Mo-CF_3_ bond, slowly appeared in the ^19^F NMR spectrum. However, the yield of the product as it was measured in respect to an C_6_H_5_CF_3_ internal standard, was less than 5%. As the reaction progressed, the product peak along with the one of Cd(CF_3_)_2_(DME), (δ = −37.0 ppm, ^2^*J*_F-Cd(111)_ = 454 Hz, ^2^*J*_F-Cd(113)_ = 472 Hz), disappeared, giving rise to resonances at δ = −80.0 ppm assigned to CF_3_H (^2^*J*_F-H_ = 78 Hz), and δ = −134.9 ppm, assigned to C_2_F_4_, indicating decomposition. The reaction yield was 10%, which was subsequently improved, albeit not significantly, when carefully dried CuBr was added to the reaction mixture (Scheme 1). Attempts to isolate the new (η^7^-C_7_H_7_)Mo(CO)_2_CF_3_ (**I**) with ambient temperature workup failed, and finally complex **I** was obtained at 17% yield by purification at −20 °C. Complex **I** is stable under inert atmosphere for a few minutes. It also decomposes rapidly when in solution at temperatures higher than −20 °C.

As substitution of a CO by a PMe_3_ ligand is known to enhance the stability of a metal complex [5], the synthesis of the (η^7^-C_7_H_7_)Mo(CO)(PMe_3_)CF_3_ was targeted next. The new (η^7^-C_7_H_7_)Mo(CO)(PMe_3_)I (**III**) was synthesized, characterized, and served as a precursor for a similar to the aforementioned trifluoromethylation reaction using Cd(CF_3_)_2_(DME)/CuBr (Scheme 1). The reaction mixture was always maintained at −10 °C, and after workup in a chromatography column at the same temperature, complex **II** was isolated as a dark green solid. Upon isolation, complex **II** is stable in air for only a few minutes, and stable under inert atmosphere for a few hours. Our experiments indicated that **II** decomposes rapidly, when in solution at room temperature, or when the workup is attempted at temperatures higher than −10 °C.

**Figure 1:**
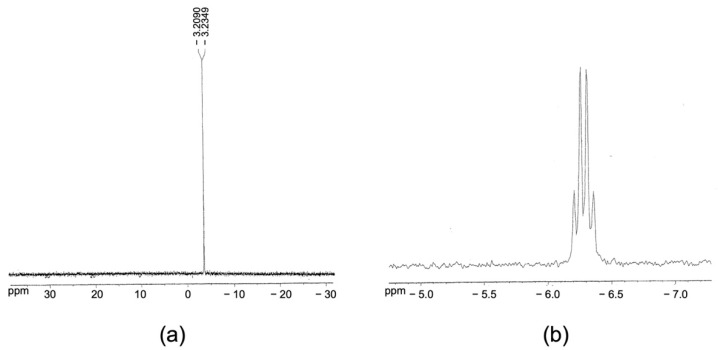
The ^19^F NMR (**a**) and ^31^P NMR (**b**) of complex **II**. Refer to the text for the analysis of the peaks and their ppm values.

Complex **II**, fluorine-to-phosphorus coupling constants, *J*_F-P_, are reported for the first time for cycloheptatrienyl complexes. In particular, the ^31^P NMR chemical shift of (η^7^-C_7_H_7_)Mo(CO)(PMe_3_)CF_3_ registers as a quartet as the ^31^P nucleus interacts with three ^19^F nuclei, with ^3^*J*_F-P_ = 8.3 Hz. The same value is recorded for the ^3^J_F-P_ of in the ^19^F NMR for the doublet observed for complex **II** (Figure 1).

## 3. Discussion

Trifluoromethyl derivatives of low-valent, late transition metal complexes are known to be more thermally and oxidatively stable than the analogous methylated species. This is believed to be due to back-bonding from the metal d orbitals into the unoccupied C-F antibonding orbitals; this bonding type is not available for M-CH_3_ analogues. However, making similar arguments for early transition metals is problematic due to insufficient data. Considering the interest in the chemistry of the CF_3_ group [14,15], as well as the intriguing instability of previously reported, methylated η^7^-cycloheptatrienyl complexes, we set out to explore the synthesis, preparation, and stability of analogous trifluoromethyl species for the first time.

An additional consideration that makes the exploration of the newly synthesized complexes attractive is the fact that the chemistry of complexes containing carbocyclic rings of higher than five hapticity is largely overlooked, most likely due to their higher instability compared to the reported stability of other cyclopentadienyl derivatives. For example, the thermal instability of the aforementioned (η^7^-C_7_H_7_)Mo(CO)_2_CH_3_ complex is in stark contrast to the high stability of the closely related (η^5^-C_5_H_5_)Mo(CO)_3_CH_3_, which is reported to be indefinitely stable at room, as well as elevated, temperatures, and can melt without decomposition.

Even though routes to L_n_MCH_3_ complexes are well-established, utilizing highly efficient, commercially available reagents, such as CH_3_Li and CH_3_MgBr, for the synthesis [16], analogous routes to of L_n_MCF_3_ derivatives are not straightforward as it is not similarly efficient and easy to access trifluoromethylating reagents. This results in relatively small L_n_MCF_3_ complexes compared with L_n_MCH_3_ ones [16].

Complex **I** appears to be more thermally stable than its methyl counterpart (η^7^-C_7_H_7_)Mo(CO)_2_CH_3_, as an isolated solid and as its workup is performed at much higher temperatures. This fact can be explained by the stabilization of the Mo-C bond resulting from the back donation of the π* orbital of the trifluoromethyl group. As it has been previously indicated, the substitution of a trifluoromethyl group for a methyl group increases the thermal stability of low valent transition metal complex by raising the decomposition temperature of the perfluorinated derivatives by 75–100 °C. An illustrative literature example is the trifluoromethyl cobalt derivative CF_3_Co(CO)_4_, which does not decompose when heated to 90°, whereas the CH_3_Co(CO)_4_ decomposes near −35 °C [3]. Regardless the increased stability, complexes **I** and **II** are shown to still be highly thermally unstable, especially when they compared (in a similar manner that the methyl analogs are compared) with the closely related known, cyclopentadienyl complexes, (η^5^-C_5_H_5_)Mo(CO)_3_CF_3_ and (η^5^-C_5_H_5_)Mo(CO)_2_(PMe_3)_CF_3_. The C_5_H_5_-Mo complexes are highly stable both in the solid form and in solution, with the former reported not to decompose for two years in the absence of light. Comparison of the IR C-O stretches (2020 and 1937 cm^−1^ for the (η^5^-C_5_H_5_)Mo(CO)_3_CF_3_ vs. 2007, 1953 cm^−1^ for the (η^7^-C_7_H_7_)Mo(CO)_2_CF_3_) may suggest that the cycloheptatrienyl ligand may donate more electron density than the η^5^-C_5_H_5_ ligand through the Mo center into the π* orbitals of the carbonyl and trifluoromethyl ligands, in a manner of making the trifluoromethyl group of complex **I**, weaker, and thus more reactive than the trifluoromethyl group of (η^5^-C_5_H_5_)Mo(CO)_3_CF_3_. This is consistent with the formal charges of C_7_H_7_^+^ and C_5_H_5_^-^ [8]. Nevertheless, the latter and complex **I** differ not only in terms of the carbocyclic group, but also by the fact that I has one less carbonyl substituent, a fact that makes unclear the direct comparison of the effect of the carbocyclic group on the M-CF_3_ bond.

Comparison of the stability of complexes **I** and **II** indicates that the phosphine analog is more stable than the di-carbonyl one, a trend which has been previously reported for methylated molybdenum derivatives.

Conclusively, the preparation of the first cycloheptatrienyl molybdenum complexes with a M-CF_3_ bond can lead to useful insights of the behavior of the trifluoromethyl group in these environments by future reactivity as well as structural studies, similar to the ones that have been conducted for the methylated analogs.

## 4. Materials and Methods

All reactions and manipulations were performed in oven- or flame-dried glassware under nitrogen atmosphere, either in a glovebox or by using standard Schlenk techniques. Column chromatography was carried out using silica gel 62 (60–200 mesh) supplied by Mallinckrodt SilicAR (Mallinckrodt Pharmaceuticals, Staines-upon-Thames, UK) which had been pre-dried at 250 °C under high vacuum. Furthermore, 1,2-dimethoxyethane (DME), diethyl ether (Et_2_O) and toluene (C_6_H_5_CH_3_) were distilled from sodium- benzophenone ketyl under N_2_ atmosphere. Hexanes (C_6_H_12_) and C_6_D_6_ were distilled from sodium (Na). Dichloromethane (CH_2_Cl_2_) and d-chloroform (CDCl_3_) were dried over calcium hydride (CaH_2_) and distilled under nitrogen prior to use. Copper bromide (CuBr) was purchased from Aldrich and dried by heating to 45 °C for 12 h under high vacuum. Trimethylphosphine (PMe_3_) was purchased from Aldrich and used as received. (C_7_H_7_)Mo(CO)_2_I, and Cd(CF_3_)_2_(DME), were prepared and purified by literature methods [17,18].

The ^1^H NMR, ^13^C NMR, ^31^P NMR, and ^19^F NMR spectra were recorded on a Bruker either DRX 400 or DRX 500 spectrometers with Nalorac BB probes. All ^1^H chemical shifts are reported in ppm (δ) relative to SiMe_4_. ^19^F NMR and ^31^P NMR chemical shifts are given in ppm upfield from CFCl_3_ and H_3_PO_4_ (85% aqueous solution), respectively. Multiplicities are indicated by s (singlet), d (doublet), t (triplet), q (quartet). Coupling constants are reported in Hertz (Hz). Infrared spectra (IR) were recorded on an ATI Mattson Genesis Series FTIR spectrometer and are given in cm^−1^. LRMS-EI were also obtained on a Hewlett Packard 5987A quadrupole instrument. HRMS analyses were performed using a Finnigan LCQ spectrometer in the APCI mode. Elemental combustion analyses were performed by Midwest Microlab, LLC of Indianapolis, IN, USA.

***Syntheses of (η^7^-C_7_H_7_)Mo(CO)_2_(CF_3_) (I)*** (C_7_H_7_)Mo(CO)_2_I (50 mg, 0.135 mmol), Cd(CF_3_)_2_(DME) (92 mg, 0.270 mmol) and CuBr (40 mg, 0.279 mmol) were loaded into a 10 mL round bottom flask and the apparatus was attached to a vacuum line. It was then cooled to −196 °C and evacuated. Anhydrous DME (4 mL) was condensed into the flask and the reaction mixture was allowed to reach −20 °C with stirring. After 5 h at that temperature, the reaction flask was opened, and the contents were filtered. The filtrate was chromatographed at −20 °C, on an alumina column (15 × 1 cm) using dry diethyl ether as eluent. A green fraction was collected, its volume was reduced to about 1 mL. Hexanes (8 mL) were added, and the solution was placed in a −78 °C bath for 2 h, to precipitate a yellow-green solid identified as **I** (7 mg, 0.022 mmol, 16% yield). ^1^H NMR (δ ppm, in CDCl_3_): 5.50 (s, 7H, C_7_**H_7_**); ^13^C NMR (δ ppm in CDCl_3_): 93.4 (s, 7C, **C_7_**H_7_,); ^19^F NMR (δ ppm, in C_6_D_6_): −0.2 (s, 3F, C**F_3_**). FT-IR (cm^−1^, KBr pellet) 2009, 1954 (C-O), 1055, 957 (C-F).

***Synthesis of (η^7^-C_7_H_7_)Mo(CO)(PMe_3_)I (III)*** A stirred (C_7_H_7_)Mo(CO)_2_I (300 mg, 0.811 mmol) solution in toluene (20 mL) was treated with PMe_3_ (0.8 mL, 1 M solution in toluene). The color of the solution immediately changed from green to orange and upon heating to 85°C the solution turned from black to green. After stirring at this temperature, the solution for 1 h, the reaction was stopped by cooling down to room temperature and the solution was filtered. All the volatiles were removed under vacuum and the remaining green solid was recrystallized from dichloromethane–hexane to give **III** (0.241 mg, 71% yield). ^1^H NMR (δ ppm, in CDCl_3_): 1.53 (d, 9H, P**(**C**H_3_)_3_**, ^3^*J*_P-H_ = 8 Hz); 5.15 (s, 7H, C_7_**H_7_**); ^13^C NMR (δ ppm in CDCl_3_): 19.8 (d, 3C, P(**C**H_3_)**_3_**, ^1^*J*_C-P_ = 27 Hz), 90.7 (s, 7C, **C_7_**H_7_); ^31^P{^1^H} NMR (δ ppm in CDCl_3_): 30.9 (s, 1P, **P**(CH_3_)_3_). FT-IR (cm^−1^, KBr pellet) 1997, 1945 (C-O). LRCI-MS (m/e, ion, %): (C_7_H_7_)Mo(CO)(PMe_3_)I, 418, 23; (C_7_H_7_)Mo(PMe_3_)I, 390, 10; (C_7_H_7_)Mo(CO)I, 342, 29; (C_7_H_7_)MoI, 314, 19; (C_7_H_7_)Mo, 187, 69, (C_7_H_7_), 91, 100. Measured HRCI-MS m/z: 419.9151, Calculated: 419.9037 (Δ m/m = 2.7 ppm). Elemental Analysis Found: C 31.39, H 3.88. Calc: C 31.60, H 3.86.

***Syntheses of (η^7^-C_7_H_7_)Mo(CO)(PMe_3_)CF_3_ (II)*** (C_7_H_7_)Mo(CO)(PMe_3_)I (30 mg, 0.072 mmol), Cd(CF_3_)_2_(DME) (40 mg, 0.117 mmol), and CuBr (30 mg, 0.209 mmol) were loaded into a 10 mL round bottom flask and the apparatus was attached to a vacuum line. It was cooled to −196 °C and evacuated. Anhydrous DME (4 mL) was condensed into the flask and the reaction mixture was allowed to reach −10 °C with stirring. After 5 h at that temperature, the reaction flask was opened, and the contents were filtered. The filtrate was chromatographed at −10 °C, on an alumina column (15 × 1 cm) using Et_2_O as eluent. A green fraction was collected, and its volume was reduced to 1 mL, Hexanes (8 mL) were added, and the solution was placed in a −78 °C bath for 2 h, to precipitate a yellow-green solid identified as **II** (14 mg, 0.039 mmol, 54% yield). ^1^H NMR (δ ppm, in CDCl_3_): 1.47 (d, 9H, P**(**C**H_3_)_3_**, ^3^*J*_P-H_ = 7 Hz), 5.13 (s, 7H, C_7_**H_7_**); ^13^C NMR (δ ppm in CDCl_3_): 18.6 (d, 3C, P(**C**H_3_)**_3_**, ^1^*J*_C-P_ = 24 Hz), 90.9 (s, 7C, **C_7_**H_7_,); ^31^P{^1^H} NMR (δ ppm in CDCl_3_): −6.3 (q, 1P, **P**(CH_3_)_3_, ^3^*J*_P-F_ = 8.3 Hz); ^19^F NMR (δ ppm, in C_6_D_6_): −3.2 (d, 3F, C**F_3_**, ^3^*J*_F-P_ = 8.3 Hz). FT-IR (cm^−1^, KBr pellet) 2020 (C-O), 1054, 1077 (C-F). Elemental Analysis Found: C 40.62, H 5.49. Calc: C 40.02, H 4.48.

## Data Availability

All data reported herein are accompanying the present article.
